# Intrapericardial Dialysis Catheter: A Rare Cause of Cardiac Tamponade Emphasizing the Importance of Early Diagnosis and Management

**DOI:** 10.7759/cureus.89344

**Published:** 2025-08-04

**Authors:** Simon Landsweerdt, Simon Bertrand, Chloé Halbrecq, Thibault Gennart, Philippe Eucher

**Affiliations:** 1 Department of Cardiovascular and Thoracic Surgery, Mont-Godinne University Hospital, Yvoir, BEL; 2 Departement of Intensive Care, Mont-Godinne University Hospital, Yvoir, BEL

**Keywords:** cardiac tamponade, dialysis catheter malposition, emergency sternotomy, hemodialysis, transthoracic contrast echocardiography

## Abstract

We report a rare and serious case of intrapericardial malposition of a dialysis catheter in a 70-year-old patient with chronic kidney disease secondary to IgG kappa amyloidosis. The complication was initially revealed by an episode of supraventricular arrhythmia and confirmed through imaging studies. Catheter removal led to hemodynamic decompensation due to a compressive pericardial effusion, which required emergency sternotomy for drainage. The postoperative course was favorable. This case highlights the importance of thorough assessment of catheter placement and the need for prompt, multidisciplinary management in rare but potentially fatal complications of hemodialysis.

## Introduction

Subclavian vein cannulation for hemodialysis was first described in 1969 [1]. It is now a widely used technique for temporary vascular access in hemodialysis. Effective hemodialysis depends on reliable vascular access, which is often initially achieved through the insertion of a tunneled central venous catheter (CVC). In typical cases, catheter placement is uneventful, and complications, when they occur, are most often related to insertion rather than removal. Although catheter placement under fluoroscopic guidance is generally considered safe, CVC-associated cardiac tamponade remains a rare but life-threatening complication, with an incidence of less than 1% [2,3].

We present an exceptional case of intrapericardial malposition of a dialysis catheter, which led to cardiac tamponade not at the time of insertion but following its removal, in the context of complex comorbidities. This case highlights the diagnostic and management challenges of a rare complication and reinforces the need for surgical backup when catheter malposition is suspected.

## Case presentation

A 70-year-old male was admitted for rapidly worsening renal function in the context of end-stage chronic kidney disease secondary to IgG kappa amyloidosis. His medical history included two squamous cell carcinomas (oropharyngeal and nasal cavity), treated five and three years prior with chemoradiotherapy, type 2 diabetes, and active smoking.

**Figure 1:**
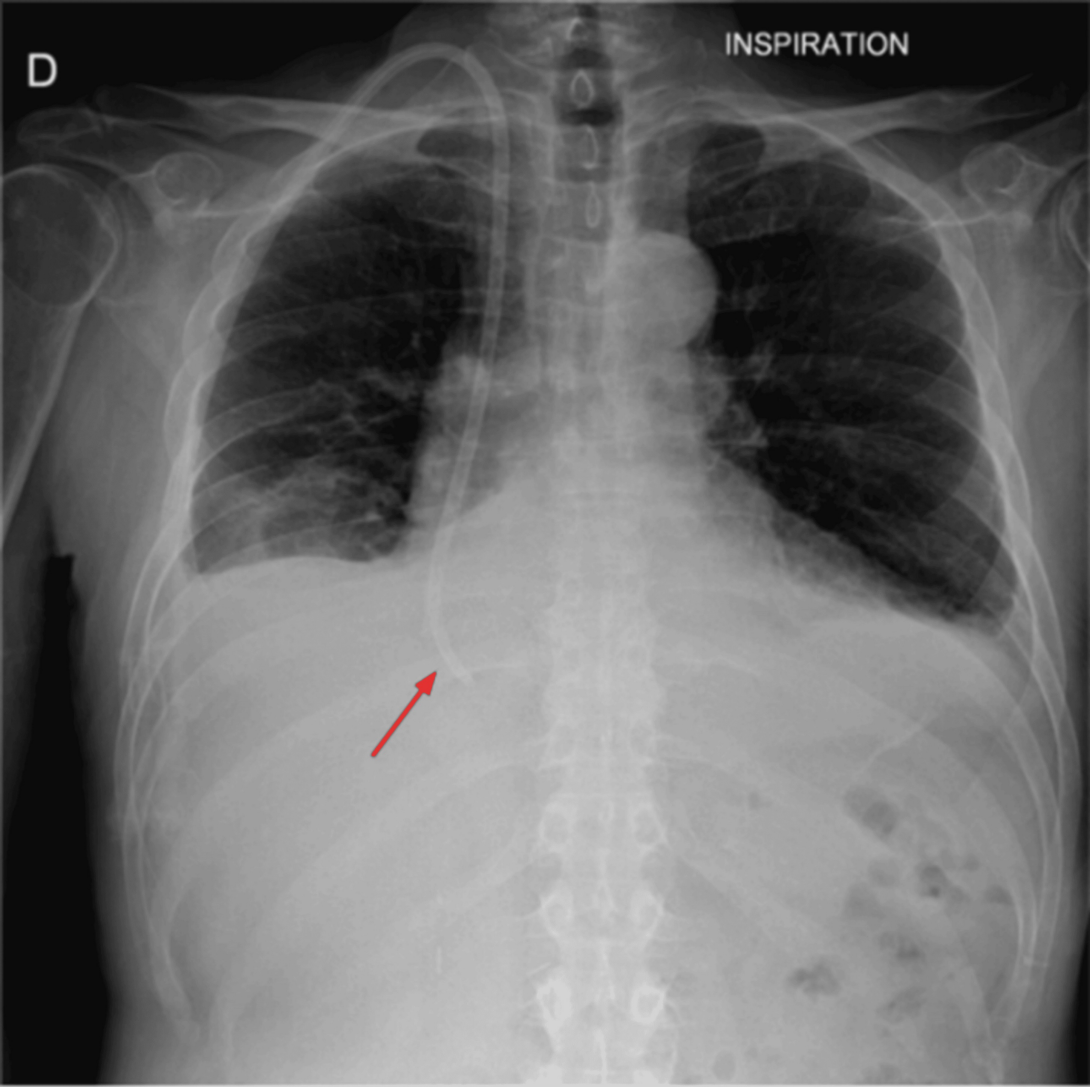
Post-procedural chest radiography. Post-procedural chest radiograph confirming intracardiac placement of the catheter (red arrow).

Two days prior to admission, a right kidney biopsy was performed as part of the etiological workup and was complicated by a stable right renal hematoma. In the setting of deteriorating renal function, a tunneled dialysis catheter was urgently placed via the right internal jugular vein under fluoroscopic guidance without immediate complication; blood return was satisfactory (Figure 1).

**Figure 2:**
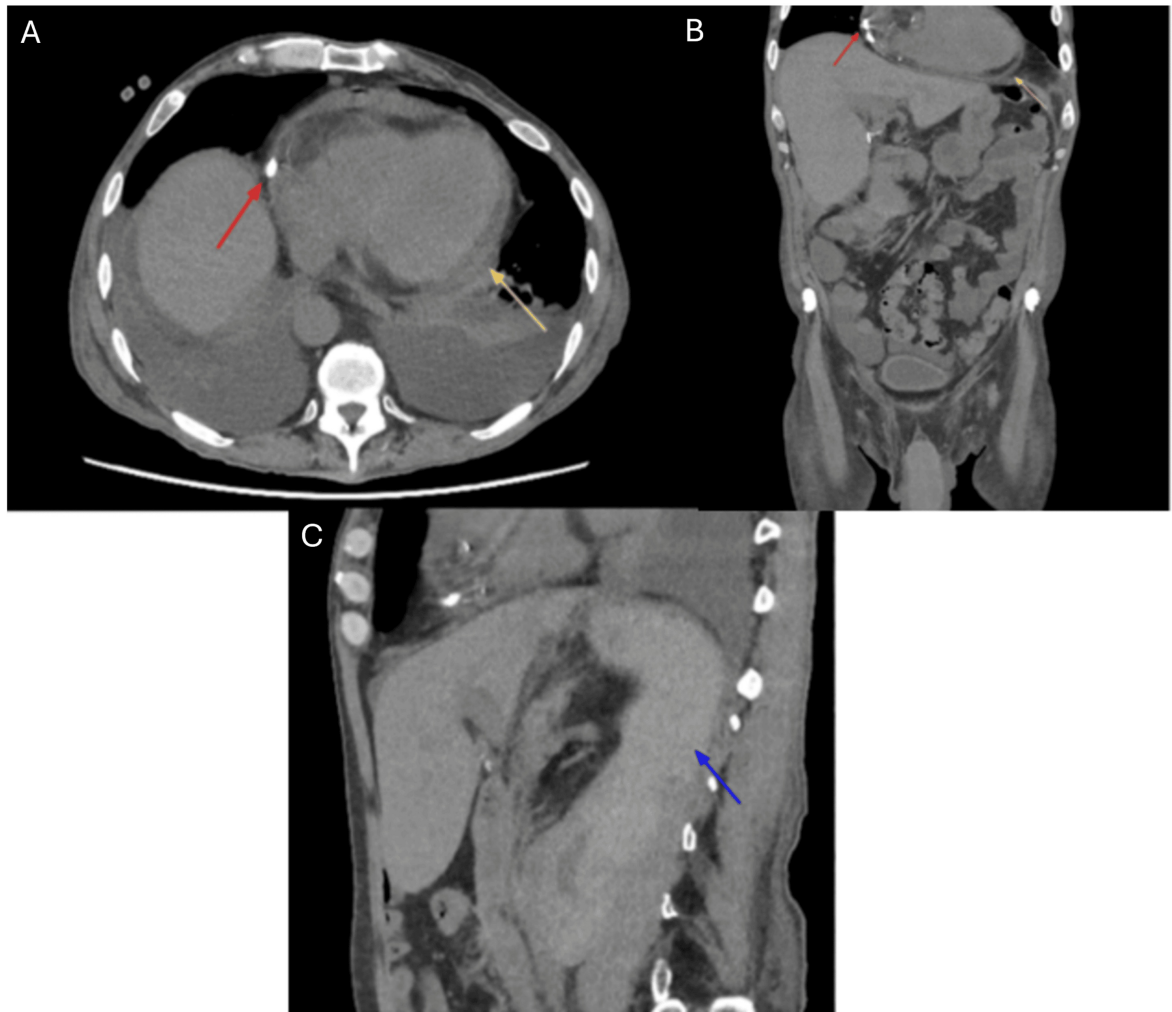
CT scan revealing intrapericardial malposition of the catheter. (A-C) CT scan showing intrapericardial malposition of the catheter (red arrow) associated with minimal pericardial effusion (yellow arrow). The retroperitoneal hematoma (blue arrow) appears stable. (A) Axial view; (B) Coronal view; (C) Sagittal view.

**Figure 3:**
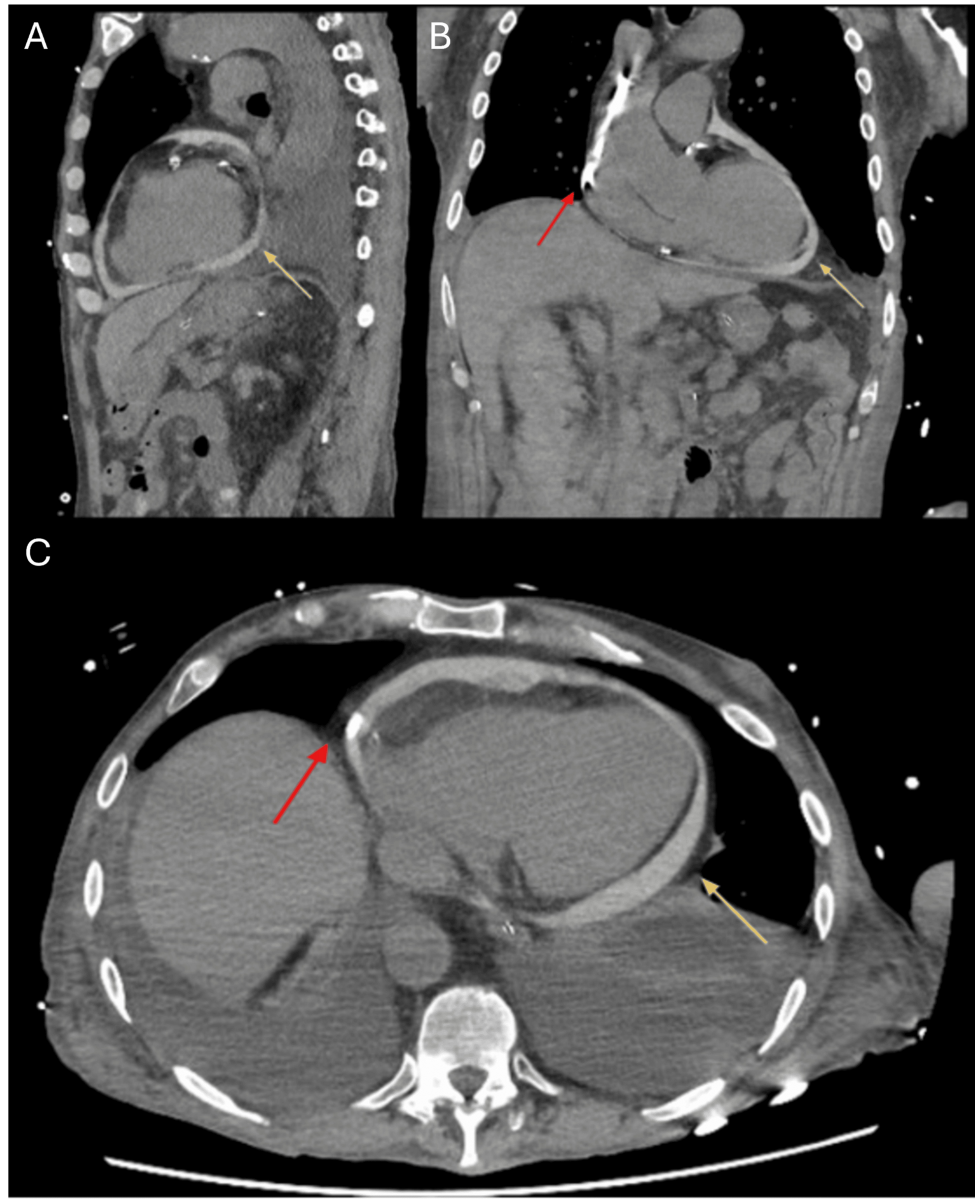
Contrast-enhanced CT via the catheter. (A-C) Contrast-enhanced CT via the catheter (red arrow) showing an increase in pericardial effusion (yellow arrow) compared to the previous scan. (A) Sagittal view; (B) Coronal view; (C) Axial view.

Three hours post-procedure, the patient developed new-onset atrial fibrillation with rapid ventricular response, prompting admission to the intensive care unit. An abdominal CT scan confirmed a stable renal hematoma but unexpectedly revealed the catheter tip within the pericardial space (Figure 2). Transthoracic echocardiography showed a small non-compressive pericardial effusion without clear catheter visualization. A contrast-enhanced CT via the dialysis catheter demonstrated contrast diffusion into the pericardial effusion, confirming intrapericardial placement (Figure 3). Notably, the patient presented no classic signs of cardiac tamponade such as chest pain, dyspnea, hypotension, or elevated jugular venous pressure. This highlights how the absence of typical clinical features can delay recognition and underscores the need for a high index of suspicion in at-risk patients.

**Figure 4:**
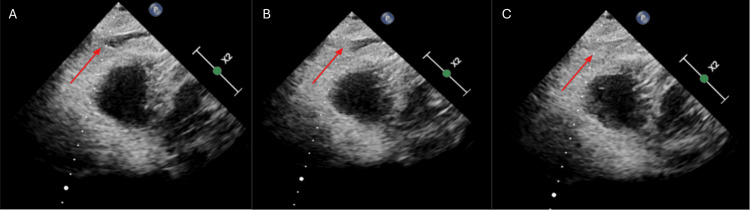
Transthoracic contrast echocardiography following microbubble injection through the catheter. (A-C) Transthoracic contrast echocardiography following microbubble injection through the catheter at three different time points. (A) Initial echocardiographic image shows no microbubble presence in the pericardial space; the red arrow indicates the expected pericardial region. (B) Intermediate frame reveals early appearance of microbubbles near the pericardium, indicated by the red arrow. (C) Clear accumulation of microbubbles in the pericardial space, highlighted by the red arrow, confirming pericardial leakage.

The patient was transferred to our center with cardiac surgery facilities. Under general anesthesia, the catheter was cautiously withdrawn by seven centimeters without immediate hemodynamic changes. Orotracheal intubation was impossible due to post-radiation trismus and was achieved nasally with fiberoptic assistance. A trial dialysis session via the repositioned catheter failed. Transthoracic contrast echocardiography with microbubble injection through the catheter confirmed contrast passage into the pericardial space without right ventricular opacification, indicating extracardiac perforation (Figure 4).

Surgical catheter removal was scheduled. Despite a cautious, planned approach, catheter removal precipitated immediate hemodynamic collapse, underscoring the unpredictability and severity of this complication. A subxiphoid pericardial drainage attempt (Marfan technique) failed, necessitating emergency sternotomy. A large compressive pericardial effusion was evacuated, leading to rapid stabilization. The fluid was grossly hemorrhagic rather than serous, consistent with a traumatic origin. No myocardial injury was found. A Jackson-Pratt drain was placed near the right heart chambers, and two 28 Fr pleural drains were inserted. A femoral catheter was placed for ongoing dialysis. Prophylactic IV cefazolin was administered for 24 hours.

**Figure 5:**
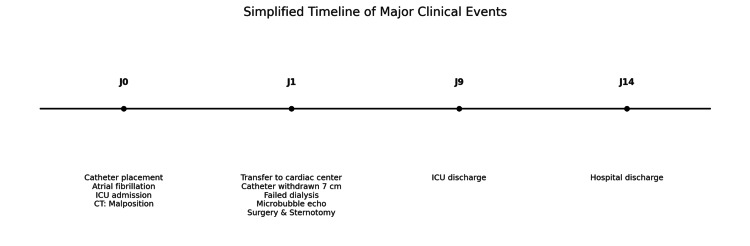
Simplified timeline of major clinical events.

The postoperative course was favorable. Pleural drains were removed on postoperative day four and the pericardial drain on day eight. A transthoracic echocardiogram performed after the removal of the pericardial drain showed no residual effusion. Dialysis resumed via the femoral catheter without complications. The patient was discharged from intensive care on postoperative day nine and from nephrology four days later. He remains under nephrology and oncology follow-up, with no need for surgical follow-up (Figure 5).

## Discussion

Tunneled dialysis catheters are frequently used in patients with chronic kidney disease requiring urgent hemodialysis. Although generally considered safe, their placement can occasionally lead to rare but life-threatening complications, such as cardiac or vascular perforation with subsequent pericardial tamponade [4,5].

Central venous catheter (CVC) malposition and vascular perforation are multifactorial complications that may arise due to anatomical variations, excessive advancement of the catheter or guidewire, and technical errors during insertion, particularly in the absence of real-time imaging. Documented risk factors include left-sided venous access, patient movement during the procedure, limited operator experience, and certain patient morphologies such as obesity or significant anatomical distortion, all of which are associated with an increased likelihood of catheter misplacement or iatrogenic vessel injury [6,7,8]. In our case, the perforation appears to have occurred immediately, despite an uneventful procedure. Several contributing factors may have increased the risk, including vascular fragility due to systemic amyloidosis, a recent renal biopsy complicated by hematoma, and a complex oncologic history that precluded transesophageal echocardiographic assessment.

Similar cases in the literature describe perforations involving the right atrium or coronary sinus, often leading to rapidly progressive tamponade [3,4,6]. Delayed migration has also been reported, sometimes occurring days or weeks post-insertion [6]. In our case, the onset of atrial fibrillation three hours post-procedure prompted imaging that suggested catheter malposition.

Confirmation was achieved using contrast echocardiography with microbubble injection through the catheter, which demonstrated passage into the pericardial space without visualization in the cardiac chambers. Although rarely reported, this technique offers high diagnostic value, especially when transesophageal echocardiography is not feasible [9,10]. Although fluoroscopy was used during the initial procedure, it failed to detect the malposition. This underscores the limitations of fluoroscopy in assessing catheter tip position in three dimensions and in distinguishing intracardiac from extracardiac locations. These limitations may be further amplified in patients with distorted thoracic anatomy, low soft-tissue contrast, or when the catheter lies parallel to the imaging plane. Importantly, if contrast had been injected through the catheter during the initial fluoroscopic assessment, it may have revealed extravascular spread and allowed earlier detection of the malposition, potentially preventing the complication [11].

Literature strongly advises against removing a malpositioned catheter outside of a fully equipped cardiac surgical setting [5,6]. Several cases have documented tamponade or cardiac arrest upon catheter withdrawal, requiring emergency surgical drainage [3,6]. Despite a cautious approach in our patient, sudden hemodynamic collapse occurred during catheter removal, necessitating an emergent sternotomy. No overt myocardial breach was found, suggesting a subtle but clinically significant erosion.

Additionally, airway management was complicated by prior head and neck cancer. Nasal fiberoptic intubation was required, further underscoring the importance of multidisciplinary coordination in managing such high-risk cases.

Preventing intrapericardial catheter misplacement requires a combination of technical vigilance and clinical suspicion. First, strict adherence to ultrasound-guided puncture and real-time fluoroscopy during catheter advancement is essential. Particular attention should be paid to the final position of the catheter tip: it should ideally rest at the cavoatrial junction, not deep within the right atrium. Post-procedural chest X-ray, although routine, may not reliably detect malposition, especially in cases of extracardiac perforation. Therefore, any unexpected arrhythmia, chest discomfort, or dyspnea shortly after catheter placement should trigger immediate echocardiographic evaluation. If doubt persists, contrast injection through the catheter, combined with echocardiography (bubble test or CT), can help confirm correct intravascular positioning. Finally, in high-risk patients, such as those with vascular fragility (amyloidosis, prior radiation, coagulopathy), consideration should be given to alternative access strategies or to performing the procedure in a hybrid suite with immediate surgical backup [12].

## Conclusions

This case highlights the rarity and severity of a potentially fatal complication associated with dialysis catheter placement. It underscores the diagnostic value of multimodal imaging, particularly transthoracic contrast echocardiography with microbubbles, and emphasizes the need for heightened vigilance and multidisciplinary coordination when intracardiac malposition is suspected. Whenever intrapericardial catheter malposition is suspected, catheter removal should be avoided outside of a cardiac surgical unit due to the high risk of sudden tamponade.
